# Disruption of *AP1S1*, Causing a Novel Neurocutaneous Syndrome, Perturbs Development of the Skin and Spinal Cord

**DOI:** 10.1371/journal.pgen.1000296

**Published:** 2008-12-05

**Authors:** Alexandre Montpetit, Stéphanie Côté, Edna Brustein, Christian A. Drouin, Line Lapointe, Michèle Boudreau, Caroline Meloche, Régen Drouin, Thomas J. Hudson, Pierre Drapeau, Patrick Cossette

**Affiliations:** 1 McGill University, Montréal, Québec, Canada; 2 Genome Quebec Innovation Centre, Montréal, Québec, Canada; 3 Centre d'excellence en neuromique de l'Université de Montréal, CHUM Research Center–Notre Dame Hospital, Université de Montréal, Montréal, Québec, Canada; 4 Department of Pathology and Cell Biology, Faculté de médecine and Groupe de recherche sur le système nerveux, Université de Montréal, Montréal, Québec, Canada; 5 Department of Dermatology, Centre Hospitalier Régional Grand-Portage, Rivière-du-Loup, Québec, Canada; 6 Department of Pediatrics, Faculty of Medicine and Health Sciences, Université de Sherbrooke, Sherbrooke, Québec, Canada; 7 Ontario Institute for Cancer Research, Toronto, Ontario, Canada; Medical Research Council Human Genetics Unit, United Kingdom

## Abstract

Adaptor protein (AP) complexes regulate clathrin-coated vesicle assembly, protein cargo sorting, and vesicular trafficking between organelles in eukaryotic cells. Because disruption of the various subunits of the AP complexes is embryonic lethal in the majority of cases, characterization of their function *in vivo* is still lacking. Here, we describe the first mutation in the human *AP1S1* gene, encoding the small subunit σ1A of the AP-1 complex. This founder splice mutation, which leads to a premature stop codon, was found in four families with a unique syndrome characterized by mental retardation, enteropathy, deafness, peripheral neuropathy, ichthyosis, and keratodermia (MEDNIK). To validate the pathogenic effect of the mutation, we knocked down *Ap1s1* expression in zebrafish using selective antisens morpholino oligonucleotides (AMO). The knockdown phenotype consisted of perturbation in skin formation, reduced pigmentation, and severe motility deficits due to impaired neural network development. Both neural and skin defects were rescued by co-injection of AMO with wild-type (WT) human *AP1S1* mRNA, but not by co-injecting the truncated form of *AP1S1*, consistent with a loss-of-function effect of this mutation. Together, these results confirm *AP1S1* as the gene responsible for MEDNIK syndrome and demonstrate a critical role of *AP1S1* in development of the skin and spinal cord.

## Author Summary

We describe a novel genetic syndrome that we named MEDNIK, to designate a disease characterized by mental retardation, enteropathy, deafness, peripheral neuropathy, ichthyosis and keratodermia. This syndrome was found in four French-Canadian families with a common ancestor and is caused by a mutation in the *AP1S1* gene. This gene encodes a subunit (σ1A) of an adaptor protein complex (AP-1) involved in the organisation and transport of many other proteins within the cell. By using rapidly developing zebrafish embryos as a model, we observed that the loss of this gene resulted in broad defects, including skin malformation and severe motor deficits due to impairment of spinal cord development. By expressing the human *AP1S1* gene instead of the zebrafish *ap1s1* gene, we found that the normal human *AP1S1* gene could rescue these developmental deficits but not the human *AP1S1* gene bearing the disease-related mutation. Together, our results confirm *AP1S1* as the gene responsible for MEDNIK syndrome and demonstrate a critical role of *AP1S1* in the development of the skin and the spinal cord.

## Introduction

Protein trafficking between organelles in eukaryotic cells is mainly mediated by clathrin-coated vesicles and their assembly requires adaptor protein (AP) complexes [1],[2]. The AP complexes also determine protein cargo selection for transport between the trans-Golgi network (TGN), endosomes, lysosomes and the plasma membrane [3],[4] and clathrin is important in establishing the basolateral domain [5]. Four ubiquitous AP complexes (AP 1–4) have been characterized and each of them is composed of four subunits. The large subunits (α, γ, δ or ε and β1–4) mediate binding to the target membrane and clathrin recruitment. The small subunit σ is part of the AP complex core and has been suggested to contribute to the stabilization of the complex, in conjunction with the medium subunit μ, which is primarily involved in protein cargo sorting [3]–[6]. Although the molecular understanding of the role of AP complexes in vesicular transport is progressing rapidly, the evidence for their role *in vivo* and in disease is more limited [4]–[7]. Knockdown or knockout of various AP-complex subunits has been attempted in different animal models, including the mouse γ and μ subunits and *C. elegans* σ subunits of AP-1A [4]–[7]. However, these are all embryonic lethal, further emphasizing the importance of these complexes for appropriate development.

So far, a few but severe genetic disorders caused by mutations in genes encoding AP complex components have been described in humans. One of the most studied involves a mutation in the β3A subunit of AP-3 which underlies the Hermansky–Pudlak syndrome 2 (HPS-2) [8]. This syndrome is characterized by oculocutaneous albinism, bleeding diathesis with absence of platelet dense bodies and abnormal depositions of ceroid lipofuscin in various organs. Mutated *AP3B3A* is believed to cause abnormal formation of intracellular vesicles from the trans-Golgi network or late endosomes, and probably mistrafficking of lysosomal proteins [7],[8]. Recently, three mutations in *AP1S2*, encoding the σ1B isoform of AP-1, have been associated with X-linked mental retardation [9]. As AP-1 is associated with synaptophysin and the vesicular acetylcholine transporter, it was suggested that these mutations cause abnormal synaptic development and function.

Erythrokeratodermia variabilis (EKV) is an autosomal dominant disease characterized by erythematous lesions and hyperkeratosis caused by mutations in two epidermally expressed connexin genes, *GJB3* (Cx31) and *GJB4* (Cx30.3) [10],[11]. Because a significant proportion of EKV families do not have mutations in *GJB3* and *GJB4*, additional EKV genes remain to be identified [10]. We previously described the identification a new locus on chromosome 7q22 for an atypical form of EKV, in families with EKV lesions, as well as lamellar and erythrodermic ichthyosis (Figure S1) [12]. In addition to the skin lesions, affected individuals from these families exhibit severe psychomotor retardation, peripheral neuropathy, and sensorineural hearing loss, together with elevated very-long-chain fatty acids and severe congenital diarrhea (Table S1). Given the similarities with the more recently described CEDNIK syndrome [13], we used the related acronym MEDNIK for mental retardation, enteropathy, deafness, neuropathy, ichthyosis, and keratodermia to designate this unique syndrome. These MEDNIK families live in a relatively isolated population descended from a limited number of ancestors, and the gene responsible for this autosomal recessive syndrome was mapped by identifying a common homozygous region [12]. In this study we present a novel splice mutation in human *AP1S1*, a ubiquitously-expressed gene encoding the small subunit σ1A of AP-1, in four families with MEDNIK syndrome from the Quebec population. This founder mutation is predicted to cause the skipping of exon 3, leading to a premature stop codon at the beginning of exon 4. To further validate the *AP1S1* mutation, we knocked down native *Ap1s1* using antisense morpholino oligonucleotides (AMOs) in the developing zebrafish and examined the ability of wild-type (WT) and mutated human mRNA to rescue the developmental phenotype. Overall, our results confirm that mutation of the *AP1S1* gene causes MEDNIK syndrome and suggest a critical implication for the *AP1S1* gene in development of the skin and spinal cord.

## Results

### Identification of Mutated *AP1S1* in Individuals with MEDNIK

**Figure 1:**
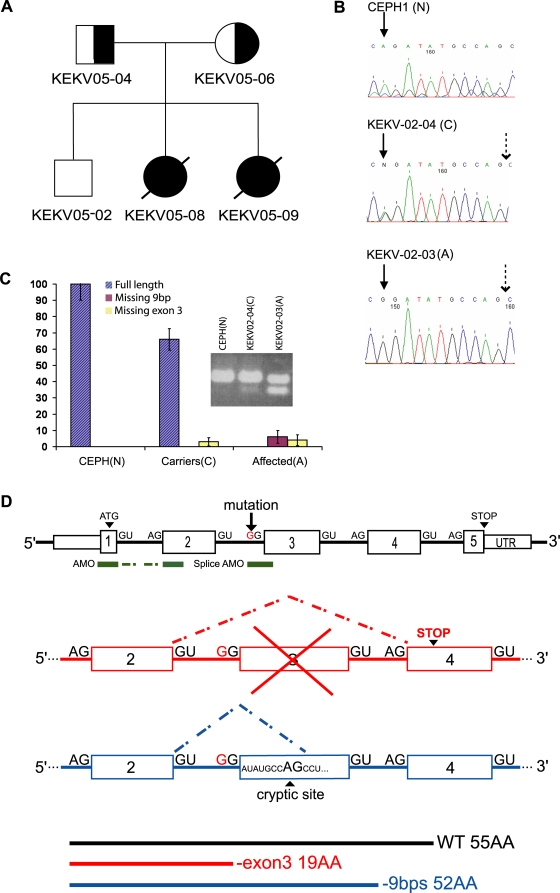
Identification and characterization of a splice mutation in *AP1S1*. A) Pedigree of the fourth MEDNIK family from the Kamouraska region. B) Sequence chromatograms of the intron 2/exon 3 junction of *AP1S1* in a normal control (N), a carrier (C) and an affected individual (A) from family EKV3-02. The filled arrow indicates the mutation (A to G) and the dotted arrow points the cryptic splice site. C) Expression of the *AP1S1* isoforms. Relative expression levels of each mRNA species (scaled to 100% of control values on the y-axis) are shown for normal controls (N; n = 2) carriers (C; n = 6) and affected individuals (A; n = 3). Values were averaged from three independent experiments. 18S RNA was used to normalize the mRNA quantity. The expression levels from this latter mRNA species could not be distinguished from the wild-type in the carriers in this figure. The expression levels are indicated as percentage of the control values Inset: RT-PCR showing the different species observed. The upper band (166 bp) contains the full-length species in both the control (N) and the carrier (C), whereas the lower band (57 bp) corresponds to the species lacking exon 3. A third mRNA species lacking 9bp, generated by the use of a cryptic splice site, was confirmed by sequencing the upper band (157 bp) of the affected individual (A). D) Schematic representation of the Human AP1S1 gene. The A>G mutation in the acceptor splice site of exon 3 predicts skipping of this exon, leading to a premature stop codon. The use of an alternative acceptor splice site within exon 3 results in a mRNA lacking 9 bps coding for an in frame protein. The location of the two different morpholinos used to knockdown *Ap1s1* in zebrafish, targeting either ATG or exon 3 acceptor splice site, are shown in green.

The region harbouring the causative gene for MEDNIK syndrome, previously named Erythrokeratodermia Variabilis type 3 (EKV3), was recently mapped to a 6.8 Mb segment of chromosome 7p using a genome-wide single nucleotide polymorphisms (SNP) panel in 3 families originating from the Bas-St-Laurent region in the province of Quebec (Canada), sharing a common ancestor at the 10th or 11th generation [12]. We genotyped a fourth pedigree, which enabled us to reduce the critical region to 5.3 Mb between markers D7S2539 and D7S518 (data not shown). Among the candidate genes mapping to that interval, *GJE1* (encoding a connexin) and *CLDN15* (encoding a claudin) were sequenced but no mutation was found. Recently, a mutation in a SNARE protein (SNAP29) was associated with cerebral dysgenesis, neuropathy, ichthyosis and palmoplantar keratoderma (CEDNIK) [13]. Since clinical manifestations of CEDNIK show striking similarities to the MEDNIK syndrome described here, we hypothesized that a mutation in *AP1S1*, a functionally related gene mapping to the candidate interval, may cause the disease. By sequencing the gene, we identified a mutation in the acceptor splice site (A to G) of exon 3 in all individuals with MEDNIK (IVS2-2A>G). This splice mutation is predicted to cause skipping of exon 3, leading to a premature stop codon at the beginning of exon 4 (Figure 1D). All parents and an unaffected sibling were heterozygous for this mutation (Figures 1B and 1C). This mutation was not observed in 180 CEPH controls.

In order to confirm the loss of exon 3, RT-PCR analyses were performed on mRNA isolated from fibroblasts using primers located in exons 2 and 4. As expected, a single band was observed in the controls. In contrast, two bands were detected in the carriers and patients (Figure 1C). Direct sequencing confirmed that the lower band corresponded to an mRNA isoform lacking exon 3. The higher band from the affected individuals corresponded to another RNA isoform, in which a cryptic splice acceptor site located 9 bp downstream of the start of the third exon was used. The resulting in frame protein is thus predicted to lack only three amino acids (Figure 1D). The full-length *AP1S1* mRNA species was not detected in these individuals. A semi-quantitative RT-PCR was performed on RNA isolated from mutation carriers and controls fibroblasts. Whereas heterozygous carriers had wild-type mRNA levels ranging form 40 to 75% of the expected value, the relative expression levels of both mutant isoforms was very low in affected individuals, corresponding to less than 10 % of the expected amount of RNA (Figure 1C). Western blot analysis of skin proteins showed faint expression of the AP1S1 protein in affected individuals, suggesting partial expression of the isoform lacking three amino acids (Figure S1C). The histological analysis of the skin revealed an epidermal hyperplasia accompanied by hypergranulosis and compact hyperkeratosis (Figure S1B).

### *Ap1s1* Knockdown in Zebrafish: Morphological Phenotype

**Figure 2:**
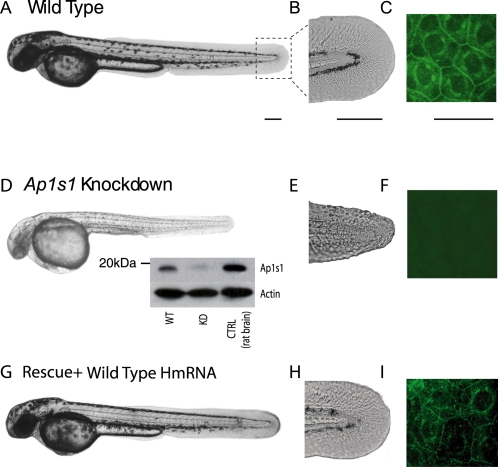
Morphological phenotype of *Ap1s1* knockdown zebrafish is rescued by over expression of human *AP1S1*. Transmitted light images of 48 hpf *Ap1s1* KD larvae show their smaller size, reduced pigmentation (D) and skin disorganization (E) compared to the WT (A, B) and the rescued larvae (G,H). Immunofluorescence of wholemount zebrafish using anti-Ap1s1 antibody showing localization of Ap1s1 to the plasma membrane (C, polygonal) and to a well defined perinuclear ring, in both normal (C) and rescued larvae (G), whereas only a residual and diffuse staining could be observed in KD larvae (F). Western blot analysis (D, inset) indicates nearly complete knockdown of Ap1s1 protein (WT = wild-type, KD = knockdown, CTRL = control rat brain proteins). To normalize the western blot analysis, proteins extracted from WT, KD and CTRL larvae were incubated with anti-actin. Scale bars in (A, D, G) = 100 µm, (B, C, E, F, H, I-Ciii) = 50 µm.

To validate whether the *AP1S1* mutation found in MEDNIK patients alters the biological function of this gene, we first knocked down *Ap1s1* in zebrafish by inhibiting mRNA translation using an AMO [14] targeting its start codon (Figure 1D). The morphological deficits of 48 hours post-fertilization (hpf) knocked down (KD) larvae (n = 68/91) are summarized in Figure 2, as the treatment was embryonic lethal at later stages. The 48 hpf *Ap1s1* KD larvae were well formed but smaller in size compared to WT, and had reduced pigmentation (Figures 2A and 2D). In addition, the KD larvae revealed prominent changes in the skin organization which were most visible in the fins (Figures 2B and 2E). In contrast to the well-defined, fan-like, ray structure of the WT caudal fin, the fin of the *Ap1s1* KD larvae was disorganized with rounded-up cells conferring a rough outline. Immature WT larvae did not show abnormal morphology of the skin and fin, suggesting that this phenotype is specific to the morpholino treatment rather than a general developmental retardation. The specificity of the AMO effect was confirmed by using Ap1s1 Western blotting and immunolabelling in wholemount larvae. With both methods we observed a decrease in the intensity of the Ap1s1-specific labeling in the *Ap1s1* KD larvae compared to the WT (Figure 2D, inset, Figures 2F and 2C). Also, larvae injected with a control AMO (5 mispaired bases) did not show significant differences compared to the WT (n = 26/26, Figure S2D). Finally, in order to mimic the splice mutation found in individuals with MEDNIK, we designed a morpholino targeting the *Ap1s1* intron 2 acceptor splice site (Figure 1D, Figure S2G, n = 32/58). In this latter experiment, we found the same abnormal skin and fin morphology as observed by using AMO targeting the *Ap1s1* start codon, although the phenotype was less penetrant.

**Figure 3:**
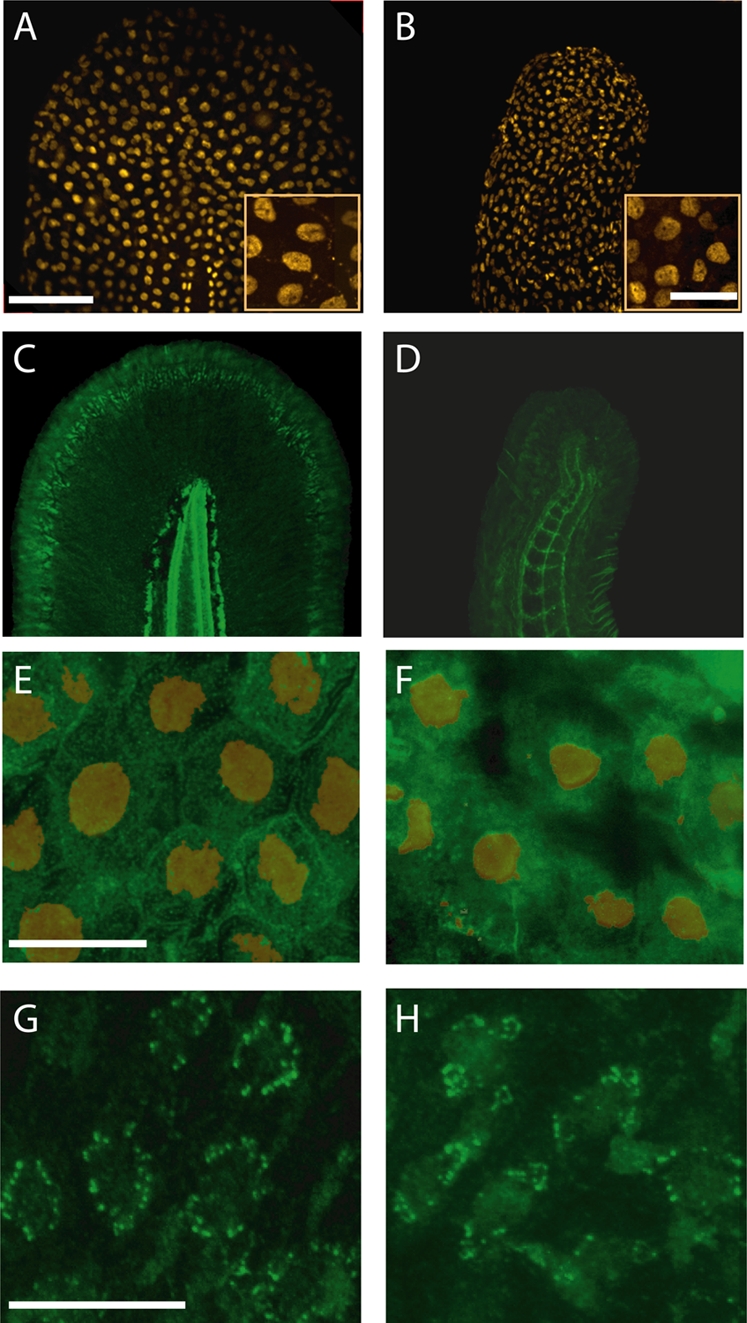
*Ap1s1* knockdown is associated with abnormal distribution of laminin and cadherin. Despite changes in the size and shape of the tail, p63-labelled nuclei (orange) of basal keratinocytes were present in KD larvae. (B). The insets show enlarged views of small groups of keratinocytes. Immunolabelling for laminin (green) in WT (C) showed normal distribution of the basement membrane along the fin fold margin, whereas in the KD larvae the residual laminin labeling was diffuse and disorganized (D). Compared to WT keratinocytes (E) labeled with p63 (orange), cadherin (green) is decreased at the cell membrane of the KD larvae (F). However, the localization of cytokeratin in WT (G) and in KD larvae (H) appeared similar. Scale bars: 10 µm, Insets in A, B, = 20 µm.

To determine if an increase in cell death underlies the skin phenotype in the KD embryo, we stained these larvae with the vital dye acridine orange [15]. We did not observe a difference compared with control (not shown), suggesting that the skin and fin disorganization was not due to an initial outgrowth followed by tissue degradation. We further tested whether the skin malformation was due to a problem in early epidermal patterning by using immunolabeling for p63, a marker of basal keratinocyte nuclei [16]. Despite the prominent changes in the size and the shape of the tail, p63-positive keratinocytes were present both in WT (Figure 3A) and KD larvae (Figure 3B). To look for a change in the population of proliferating cells, we performed immunolabelling with the phosphorylated-histone-H3 (PH3) antibody to visualize cells undergoing histone modification during mitosis, which did not reveal any obvious difference between the KD and control larvae (not illustrated). Similar results were obtained with co-immunostaining against p63 and PH3, suggesting unaffected proliferation level of basal keratinocytes population in the KD larvae (not illustrated). To further investigate whether the keratinocytes in the KD larvae exhibit specific abnormalities, we immunolabeled WT and KD larvae for laminin (Figures 3C and 3D) and for cadherin (Figures 3E and 3F). Laminins, in particular laminin 5, are synthesized by keratinocytes and are their main anchor to the basement membrane [17], while cadherins are localized to the keratinocyte cell membrane and are essential in maintaining cell-cell adhesion [18]. In the WT, laminin was detected at the outer edges of the fin (Figure 3C) while in the KD larvae (Figure 3D) the detected laminin appeared diffuse, with an abnormal localization. Furthermore, in the KD larva, cadherin immunolabeling was less obvious at the cell membrane of keratinocytes doubly-labeled with cadherin (green) and p63 (orange) (Figure 3F) In contrast, the localization of cytokeratin, a major cytoskeletal protein expressed exclusively in epithelial cells [19],[20] seemed to be preserved in KD larvae (Figures 3G and 3H).

### Behavioral Deficits and Impaired Spinal Cord Development in *Ap1s1* Knockdown Larvae

**Figure 4:**
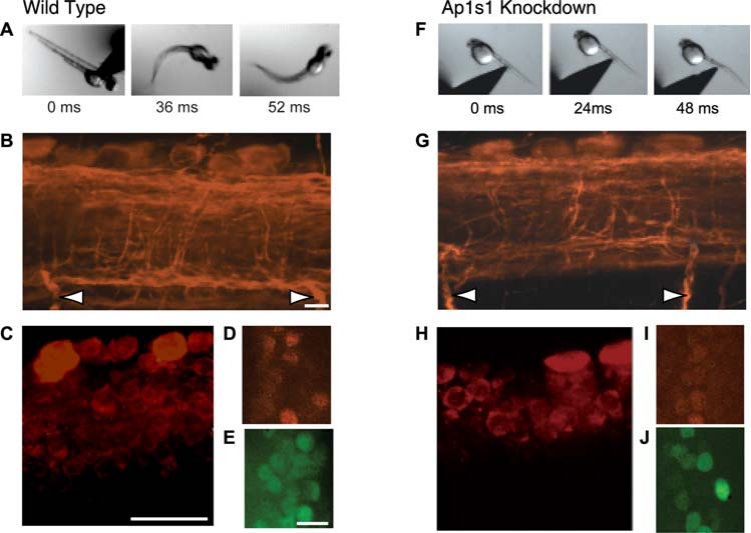
Abnormal behavioral phenotype and impaired development of spinal neural network of *Ap1s1* knockdown zebrafish. Consecutive images from films illustrating the response to touch in 48 hpf wild type (WT) (A) and *Ap1s1* KD larvae (F). The WT larva reacted to touch by swimming away. In contrast, the *Ap1s1* KD larvae exhibit slow and impaired reaction. Immunostaining in wholemount 48 hpf WT (B–E) and *Ap1s1* KD larvae (G–J) illustrating the reduced axonal labeling (anti-acetylated tubulin; B and G red, the arrows point to the ventral roots), the reduced number of newly born cells (anti-HU antibody; red in C and H), and the number of interneurons (anti-Pax2 antibody; E and J green) in the *Ap1s1* KD larvae. In turn, the number of motoneurons (anti-HB9 antibody; D and I, red) was similar both in WT and *Ap1s1* KD. Scale bars: 25 µM.

At 48 hpf WT larvae normally respond to touch by swimming, which is characterized by alternating tail movements with a beat frequency of about 30 Hz (Figure 4A) [21],[22]. In contrast, *Ap1s1* KD larvae reacted to touch by tail coils (Figure 4F), an embryonic motility pattern that usually disappears around 24 hpf [21]. Since the KD larvae exhibited severe motor impairment, we further investigated the spinal cord neural organization. An anti-acetylated tubulin staining revealed a reduction in axonal processes in the spinal cord of *Ap1s1* KD larvae (Figure 4G) compared to the WT (Figure 4B). To quantify the number of newly born neural cells, wholemount 48 hpf larvae were labeled using anti-HU, as this RNA binding protein is found in neuronal cells leaving the mitotic cycle [23]. The number of newly born neurons in KD larvae (Figure 4H, n = 3, 41±3) significantly decreased to 51% of control, WT, levels (Figure 4C , n = 3, 81±9, p<0.001). We also quantified the progenitor population in the spinal cord using an anti-PH3, but we did not find a significant change between *Ap1s1* KD and control larvae groups (n = 6 each, not illustrated), nor did we observe significant cell death upon staining with acridine orange. To study which population of neurons was specifically affected, we labeled interneurons and motoneurons by using anti-Pax2, which labels a large subset of early differentiating interneurons [24] and anti-HB9, a homeobox gene necessary for motoneuron differentiation [25]. Interestingly, whereas the number of motoneurons was unchanged (Figures 4D and 4I; n = 3 each, p = 0.42), we observed a 46 % reduction in the number of interneurons in *Ap1s1* KD larvae compared to the WT (Figures 4E and 4J; n = 3 each, WT 28±1.5, KD 13±0, p<0.001). This behavioral and spinal phenotype was specific to the morpholino treatment and not just a reflection of general developmental retardation, as reflected by the sparing of motoneurons and loss of interneurons, which is not observed during normal development.

### Overexpression of Human mRNA Rescues Ap1s1 Function in AMO Larvae

All larvae co-injected with human wild type human *AP1S1* mRNA and *Ap1s1* AMO exhibited restoration of the skin organization, pigmentation (Figures 2G–I), as well as swimming behavior (n = 35/35 fish). Conversely, larvae co-injected with human *AP1S1*-exon3 mRNA and *Ap1s1* AMO showed skin and motor deficits similar to those observed in *Ap1s1* KD larvae, suggesting a loss of function of this truncated form of the protein (Figure S2F, n = 24/24). However, co-injection of the human alternative mutant *AP1S1*-9bp mRNA together with the AMO rescued the phenotype (Figure S2E, n = 19/19 fish), suggesting that this protein isoform lacking 3 amino acids remains functional. Larvae injected with the mismatch morpholino oligonucleotide were similar both morphologically and behaviorally to the WT (Figure S2D, n = 26/26).

## Discussion

In this study, we demonstrated that the autosomal recessive MEDNIK syndrome, described in the population of Quebec, is caused by a founder mutation in *AP1S1*. More specifically, we have shown that the A to G mutation in the acceptor splice site of exon 3 of *AP1S1* (IVS2-2A>G) was associated with skipping of this exon, leading to a premature stop codon. To our knowledge, this is the first report of a mutation in the human *AP1S1* gene. We also demonstrated that the IVS2-2A>G mutation produced a loss of function effect in zebrafish. These findings support the conclusion that the *AP1S1*-exon 3 mutation is indeed pathogenic. Our results are consistent with the recent description of a mutation in SNAP29, a regulator of vesicle fusion to target membrane, found in CEDNIK syndrome. Indeed, the CEDNIK syndrome shows striking similarities to MEDNIK and mutated genes in these two diseases play a role in vesicular trafficking [13].

Recently, mutations in the σ1B subunit of AP-1 (*AP1S2*) were identified in patients with X-linked mental retardation [9]. In contrast to the *AP1S1* mutation described here, these individuals do not exhibit defects in other organs. Presumably, the loss of the σ1B subunit can be compensated in tissues outside of the central nervous system. Even though the mutations found in *AP1S2* are predicted to cause premature stop codons in exons 2 and 3, it has not been determined if functional protein products were present in the affected individuals.

### *In Vivo* Characterization of AP1S1 Function

Little is known about the σ subunit role in AP complex formation and function *in vivo*. It is suggested that AP1S1 contributes to the AP complex core stabilization [6],[26]. Furthermore, in AP-1 and AP-3, the σ subunit is suggested to interact with “dileucine-based” recognition signal on cargo proteins, in combination with the γ or the δ subunit respectively. Therefore, this implicates the σ subunit in protein sorting as well [27]. However, attempts to interfere with AP1S1 function *in vivo* were not successful so far, as they resulted in embryonic lethality. Similar results were obtained by interfering with most of the other subunits of the AP-1 complex, further emphasizing its importance for appropriate development [4],[7]. In this study, we knocked down *Ap1s1* in zebrafish and were able to rescue the morphological and behavioral phenotypes observed in KD larvae by co-injecting WT human *AP1S1* mRNA, which further support the specificity of the *Ap1s1* knockdown. The remaining levels of Ap1s1 protein may explain viability in zebrafish, at least for the first 48 hours of development. However, because some of the AP complexes have overlapping function, compensation by other AP complexes cannot be excluded [6],[28]. Nevertheless, since the *Ap1s1* KD larvae exhibit severe deficits, neither residual levels of AP-1A and B, nor the activity of other AP complexes were sufficient for appropriate development of many cell types (skin, pigment and neural).

In this study, we demonstrate for the first time that disruption of an AP-1 subunit, more specifically the σ1A subunit, causes perturbation in epithelial cell development *in vivo*. The presence of p63 immuno positive basal cytokeratinocytes in the KD larva suggested that knocking down *Ap1s1* did not interfere with early epidermal patterning. The skin phenotype was not accompanied by an increased cell death or in the level of proliferating basal keratinocytes. Carney *et al.* [29] observed an increase in proliferating basal keratinocytes in zebrafish mutants suffering from severe epithelial disintegration and suggested that this phenomenon is a secondary consequence of inflammation and consequent loss of epithelial integrity. The lack of increased proliferation in our study could be explained by the presence of sufficient residual laminin to provide some anchoring for the keratinocytes, allowing the maintenance of some epithelial properties. However these residual levels of laminin appeared insufficient for appropriate basement membrane development. Interestingly, zebrafish embryos carrying a mutation in the gene encoding for laminin 5 suffer from severe deficits in fin formation due to disruption in basement membrane integrity [30]. In *Ap1s1* KD larvae, we also found an alteration in the localization of cadherin in basal keratinocytes, which was not accompanied by changes in cytokeratin localization, suggesting that this component of epithelial cells cytoskeleton remain unaffected by *AP1S1* dysfunction. Interestingly, the nature of the specific adaptor complex that recognizes the cadherin dileucine sorting motif is unknown, although AP-1 is a candidate [18]. Based on these observations, we suggest that *Ap1s1* knockdown resulted in failure to localize cadherin to the basolateral cell membrane which, together with an abnormal pattern of expression of laminin 5, lead to a loss of epidermal layer integrity.

### Behavioral Deficits and Abnormal Neural Development

The well-formed 48 hpf *Ap1s1* KD larvae showed a severe behavioral phenotype. Instead of reacting to touch by swimming, the KD larvae coiled in a motility pattern distinctive of younger embryos. Consistent with this observation, detailed examination of the spinal cord revealed an abnormal development. The extent of axonal processes was diminished and the number of newly born neurons was reduced to half of the WT levels due mainly to a decrease in the interneuron population, but not in motoneurons. Interestingly, as observed in the skin, no change was seen in the levels of neuronal progenitors in the spinal cord. There is mounting evidence that AP complexes such as AP-2 and AP-3 are implicated in neural function [31]. For example, mice with knockout of the AP-3 μ3Β subunit are susceptible to epileptic seizures because of deficient GABAergic vesicle formation and function [32]. Also, *mocha*, one of the mouse models for Hermansky-Pudlak syndrome (HPS) in which the δ subunit of AP-3 is mutated, suffer from neurological disorders [33]. The loss of AP-3 in these mice affected spontaneous and evoked neurotransmitter release in hippocampal mossy fiber synapses [34]. AP-2 is implicated in selective endocytosis and recycling of synaptic vesicles and also of receptors and transporters from the plasma membrane of nerve terminals [31],[35]. For example, internalization of α-amino-3-hydroxy-5-methylisoxazole-4-propionic acid (AMPA) receptors by binding to AP-2 is essential for N-methyl-D-aspartic acid (NMDA)-induced long-term-depression in the hippocampus and therefore to synaptic plasticity [36],[37]. In turn, little is known about AP-1 function in neurons, although it was reported to interact with synaptophysin, one of the most abundant proteins in synaptic vesicles [38], as well as with vesicular acetylcholine transporter [39]. Moreover, AP-1 binds to the ubiquitous microtubule-associated motor protein KIF13A, a member of a protein family implicated in neuronal transport of membranous organelles, synaptic vesicles and proteins from the cell body to the axons and dendrites [40],[41]. Mice with mutations in members of this protein family (KIF1A, KIF1Bβ) show reduced synaptic vesicles in the synaptic terminals and suffer from in sensory-motor deficits [42]. Also, mutations in *KIF1Bβ* cause Charcot-Marie-Tooth hereditary peripheral neuropathy type 2A in humans [42]. It is thus possible that AP1S1, in addition to its possible implication in synaptic vesicles regulation and formation, could be implicated in their transport toward the neural processes. Although not much is known about the precise role of AP-1 in the developing central nervous system, we show here that the disruption of the AP-1 function is associated with substantial perturbation of a subset of spinal interneuron differentiation.

### The Zebrafish *Ap1s1* Knockdown and Its Contribution to Understanding of the MEDNIK Syndrome

*Ap1s1* KD larvae exhibit abnormal development of neurons and skin cells, a phenotype that shows similarities to the clinical manifestations observed in individuals with MEDNIK. Based on the observation of reduced neurogenesis we have made in zebrafish, we speculate that MEDNIK syndrome in affected patients is caused by an impaired development of various neural networks, including the spinal cord (ataxia and peripheral neuropathy) and possibly the brain (microcephaly and psychomotor retardation) and inner ear (sensorineural deafness). We also hypothesize that disruption of AP1S1 in humans may be associated with more extensive perturbation of organogenesis. Indeed, growth retardation, digestive tract malformations and dysfunction (chronic diarrhea), and elevation of very long chain fatty acid observed in individuals with MEDNIK syndrome might reflect more widespread perturbation of vesicular transport and of epithelial cell development. One intriguing question is why the *AP1S1*-exon 3 mutation is not lethal in homozygous individuals with MEDNIK. Indeed, overexpression of human *AP1S1*-exon3 mRNA failed to rescue the phenotype observed in *Ap1s1* KD larvae, suggesting a loss of function of this critical protein. However, co-injection of the *AP1S1*-9bp human mutant mRNA with AMO, the alternative RNA species detected in our MEDNIK patients, rescued the phenotype, suggesting that this alternative splicing results in a functional protein. The expression of that protein isoform in patients may thus explain their viability. The fact that the *AP1S1*-9bp mRNA is expressed at low levels (less than 10 % of normal levels in fibroblasts) could explain why it is not sufficient to sustain normal development and function and further highlight the important role of AP1S1 in normal development. Furthermore, the expression levels of the different AP1S1 isoforms may vary from one tissue to another, as well as between individuals, thereby contributing to the variability of the phenotype.

Overall, these observations in zebrafish, in light of previous *in vitro* studies [31], [34], [43]–[45], suggest that AP1S1 and AP-1 complex are most likely implicated in appropriate protein sorting and transport. Interference with these pathways could therefore result in perturbation of cellular organization and be detrimental for the development of specific cell subpopulations, as we observed respectively in the skin and the spinal cord of the *Ap1s1* KD larvae. The results suggest avenues for both basic and clinical research, in order to better understand the mechanisms underlying MEDNIK and related neuro-cutaneous syndromes.

## Materials and Methods

### Patients and Biological Materials

Seventeen individuals from four families including three affected children were ascertained and examined as described [12]. Genetic material of affected individuals and unaffected siblings and parents was isolated from blood lymphocytes at Le Service de Dermatologie du CHRGP de Rivière-du-Loup and Le Service de Génétique du CHUQ (Hôpital St-François d'Assise). Fibroblast cell cultures were obtained from 3 mm punch biopsies from patients, relatives or healthy controls and were maintained in Dulbecco's Modified Eagle's medium (DMEM) supplemented with fetal calf serum 10%. The study was approved by the Institutional Review Board of the Hôpital St-François d'Assise and informed consent was obtained from all family members.

### DNA Amplification and Mutation Analysis

Coding regions of *AP1S1* were amplified by PCR from genomic DNA (primer sequences are available upon request). Total RNA was extracted from cultured primary fibroblasts harvested from skin biopsy samples using standard protocols. cDNA was prepared using random hexamers and standard procedures, and a fragment from exon 2 to exon 4 of *AP1S1* was amplified with the primers used for the Taqman exon 3 assay (see below). All DNA templates were amplified using HotStar Taq polymerase (Qiagen, Valencia, CA) and standard conditions (95°C for 5 min; 40 cycles of 95°C for 30 sec, 60°C for 30 sec and 72°C for 30 sec; and 72°C for 10 min.). Amplicons were sequenced in both directions using the same primers than for PCR.

### RT-PCR Analyses

Taqman assay was performed on cDNA (obtained from fibroblast isolated RNA) using the Taqman kit (Applied Biosystems, Foster City, CA) and according to the manufacturer's conditions. For the exon 2 assay, 300 nM of these PCR primers, AP1S1_exon2F, 5′-gagctcatgcaggttgtcct-3′; AP1S1TaqR, 5′-AGTTGAAGATGATGTCCAGCTC-3′, and 200 nM of the probe, AP1S1TaqP_exon2, 5′FAM-CCTGGAGTGGAGGGACCTCAA-TAMRA3′, were used. For the exon 3 assay, 300 nM of these PCR primers, AP1S1TaqF, 5′-TGGAGGGACCTCAAAGTTGT-3′ and AP1S1TaqR, 5′-AGTTGAAGATGATGTCCAGCTC-3′, and 200 nM of the probe, AP1S1TaqP, 5′FAM-CACACTGGAGCTGATCCACCGATAC-TAMRA3′, were used. All primers were designed using NM_001283 as the reference sequence. As an expression control for use in quantification, universal 18S primers were included in the same reaction mixes. PCR conditions were: 95°C for 10 min, 45 cycles of 30 sec at 95°C, 30 sec at 56°C, and 30 sec at 72°C. Reactions were cycled on the 7900HT Real-time PCR instrument (Applied Biosystems). Relative expression for each sample was evaluated by using the difference in the threshold cycle (ΔCt ) value to achieve a similar level of fluorescence. 18S relative expression was used to normalize for the cDNA quantity of each sample. All values correspond to an average of three independent experiments.

### Cloning of Human *AP1S1*

We designed primers (*AP1S1*-5′-TAAGCGGATCCATGATGCGGTTCATGCTATTATTC, and *AP1S1*-3′-GTAAGCCTCGAGTCAGTGGGAAAAGGGGAAAGTGG) to amplify the complete open reading frame of *AP1S1*-variant1 from a human brain cDNA library (Marathon-ready, BD Biosciences Clontech), using *Pfu* Polymerase (Stratagene). The same primers were used on patient's cDNA to get the mutated alleles, using Advantage 2 Polymerase (Clontech). By using BamHI and XhoI restriction sites introduced into the primer sequences, the PCR products was directionally cloned into pCS2+ vector. All constructs were completely sequenced to confirm the mutations, as well as to exclude any other variants that could have been introduced during the PCR amplification. Capped sense mRNAs were synthesized from pCS2+ by using the mMESSAGE mMACHINE SP6 kit (Ambion).

### Histology

Skin biopsies were also used to perform histological analysis. The samples were fixed in formalin 10% and embedded in paraffin. Sections of aproximately 5 µm were cut by using cryostat, and stained with haematoxylin and eosin.

### Morpholino Knockdown of *Ap1s1* in Zebrafish

Experiments were performed on zebrafish (*Danio rerio*) larvae raised at 28.5°C according to previously established procedures [46], and in compliance with Canada Council for Animal Care and institutional guidelines. To knockdown the function of the gene encoding for the σ1A subunit of AP-1 in zebrafish, which shares 91% identity with the human AP1S1 protein, an AMO (Gene Tools) was designed to target the initial codon of zebrafish *Ap1s1* gene (5′-ACAGAAGCATAAAGCGCATCATTTC- 3′), which differs in sequence from human *AP1S1*. In addition, a second morpholino was designed to target the acceptor splice site (intron 2) of the zebrafish *Ap1s1* gene, 5′-GACTAGCATACCTACGTAAACACAC-3′. All AMO preparation and injection procedures were according to previously described protocols [13]. The specificity of our AMO was verified by injection of a control, 5 base pairs mismatch morpholino oligonucleotide (5′-ACACAAGGATAAACCG**CAT** GATATC- 3′) as well as by Western blotting as will be described below. After establishing the AMO phenotype (1 mM), rescue experiments were preformed in which both AMO (1 mM) and human *AP1S1* WT or mutated mRNA (110 ng) were injected.

### Western Blot

Skin biopsies were obtained from normal individual, carrier and patients (lesional and non-lesional skin). The samples were frozen in liquid nitrogen and homogenized in lysis buffer (RIPA: Tris-HCl 50 mM, NaCl 150 mM, EDTA pH 8.0, Triton 1%, Sodium deoxycholate 1%, SDS 0.1%, Protease inhibitors (complete mini, Roche), Aprotinin 10 µg/ml, Leupeptine 10 µg/ml, phenylmethylsulphonyl fluoride (PMSF) 1 mM). The lysates were centrifuged at 12 000 g for 20 min at 4°C. To quantify gene knockdown, thirty 48 hpf WT and *Ap1s1* KD larvae were dechorionated and anaesthetized in 0.2% MS-222 (Sigma) and then homogenized in lysis buffer (150 mM NaCl, 1% IGEPAL CA-630, 50 mM Tris, pH 8.0, 0.5% sodium deoxycholate, 0.1% SDS. The lysates were centrifuged 10 min at 2000 g at 4°C in complete protease inhibitor cocktail (Roche). After the protein extraction, western blot protocols were the same for both human skin samples and zebrafish. The supernatants were removed and the proteins were quantified using DC protein Assay (BIO-RAD) with bovine serum albumin (BSA) as a standard. As a primary antibody, rabbit antisera DE/1 directed against Ap1s1 was used at a concentration of a 1∶5000 (antibody obtained from Dr. Traub) [47]. Horseradish peroxidase-conjugated donkey anti-rabbit IgG (1∶5000; Jackson Immunoresearch Laboratories Inc.) was used as a secondary antibody. Visualization was performed by using Western Lightning Chemiluminescence Reagent Plus (PerkinElmer). Hybridization of the same blot using anti-actin antibody was used to assess equal loading of the samples (mouse monoclonal anti-actin 1∶5000, Chemicon #MAB1501).

### Immunohistochemistry

Briefly, all dechorionated larvae were collected, anesthetized in 0.2% MS-222 (Sigma) and fixed for two hours in 4% paraformaldehyde (PFA) at room temperature as previously described [46]. Samples were then washed in phosphate buffered saline (PBS) before dehydration in 100% methanol and kept at −80°C for later use. For cytokeratin labeling, larvae were stored in Dent's fixative at –20°C Primary and secondary antibody incubations were conducted overnight at 4°C in blocking solution. Then samples were washed in PBS-Tween and incubated overnight with Alexa 488 (anti-rabbit) or 568 (anti-mouse) antibodies (Molecular Probes). After four washouts in PBS-tween, larvae were mounted on slides in glycerol 90%, for immunofluorescence imaging. Primary antibodies were used at the following dilutions: rabbit antisera DE/1 directed against Ap1s1 1∶200; monoclonal mouse anti-acetylated tubulin (Sigma) 1∶1000; monoclonal mouse anti-HB9 (Developmental Studies Hybridoma Bank 81.5C10) 1∶200; polyclonal rabbit anti-Pax2 (Covance PRB-276P) 1∶100; polyclonal rabbit anti-phosphohistone H3 (Ser10) (Upstate 06 570) 1∶100; monoclonal mouse anti-HU (Molecular Probes A21271) 1∶100; rabbit anti-laminin (Sigma L9393) 1∶100; rabbit anti-pan cadherin (Sigma C 3678) 1∶400; monoclonal mouse anti-p63 (Santa Cruz sc-8431) 1∶100; monoclonal mouse anti-cytokeratin type II KS Pan 1-8 (Progen Biotechnik 61006) 1∶10. To verify for cell death in wholemount larva, we stained them using the vital dye Acridine Orange, as described previously [48].

### Imaging

The fluorescent images represent the maximum projection of a series of 2 µm optical sections obtained in whole mount larva using a laser confocal microscope (Perkin Elmer Ultraview system mounted on a LEICA DM LFSA microscope with a 63X oil objective 1.25 NA) and Metamorph software (Universal Imaging Corp). Antibody-labeled cells (HU, BH9, PAX2 and PH3) were counted in equal length spinal cord segments (75 µm) imaged at the 14th somite and cover the entire spinal cord volume. Statistical significance between *Ap1s1* KD and WT larva groups was verified using Mann-Whitney rank sum test (Sigmastat). Transmitted light images were digitized using a digital camera (Axio Cam HRC, Zeiss) mounted on a dissecting microscope (Stem1 SV 11, Zeiss) and Axiovision 4.2 software. To document the response to touch of the 48 hpf larva high-speed video films were digitized (250 frames/sec) using a Photron Fastcam PCI high-speed video camera mounted on a Zeiss dissection microscope. The captured films were analyzed off line to determine swim frequency. Representative images from these films were used to reconstruct the movements of *Ap1s1* KD and WT larvae in Figure 4.

## Supporting Information

Figure S1 A) Typical erythrokeratodermia variabilis skin lesion. B) The histological analysis revealed an epidermal hyperplasia accompanied by compact hyperkeratosis (CH) and hypergranulosis (HG). C) Normalized western blot analysis of skin proteins indicates faint expression of Ap1s1 in affected individuals (A), probably because of partial expression of the isoform lacking three amino acids, compared to mutation carriers KEKV-02-04 (C) and control (Ctrl).Proteins were extracted from biopsies obtained from both lesional and non-lesional (*) skin in individual KEKV-01-03.
(8.78 MB TIF)

Figure S2 Representative transmitted light images of 48 hours-old zebrafish larvae illustrating the phenotype under different experimental conditions. (A) Wild type (WT), non injected larvae. (B) Knockdown larva, injected with AMO targeting the translation start site of *Ap1s1* (N-terminal). (C) Larva rescued by co-injection of AMO and human WT mRNA (HmRNA). (D) Larva injected with a mismatch AMO. (E) Larva rescued by co-injection of AMO and -9bp Human mRNA. (F) larva co-injected with AMO and -exon3 HmRNA. (G) Knockdown larva, injected with AMO targeting the acceptor splice site (intron 2) of *Ap1s1*.
(7.41 MB TIF)

Table S1 Clinical features of affected individuals with MEDNIK. EKV: erythokeratodermia variabilis; VLCFA: very long chain fatty acids; NA: not available; mo: month.
(0.03 MB DOC)
