# Supplementary figures and images for: Disruption of AP1S1, Causing a Novel Neurocutaneous Syndrome, Perturbs Development of the Skin and Spinal Cord
Source: PLoS Genet. 2008 Dec 5;4(12):e1000296. doi: 10.1371/journal.pgen.1000296 (PMC2585812; doi:10.1371/journal.pgen.1000296)

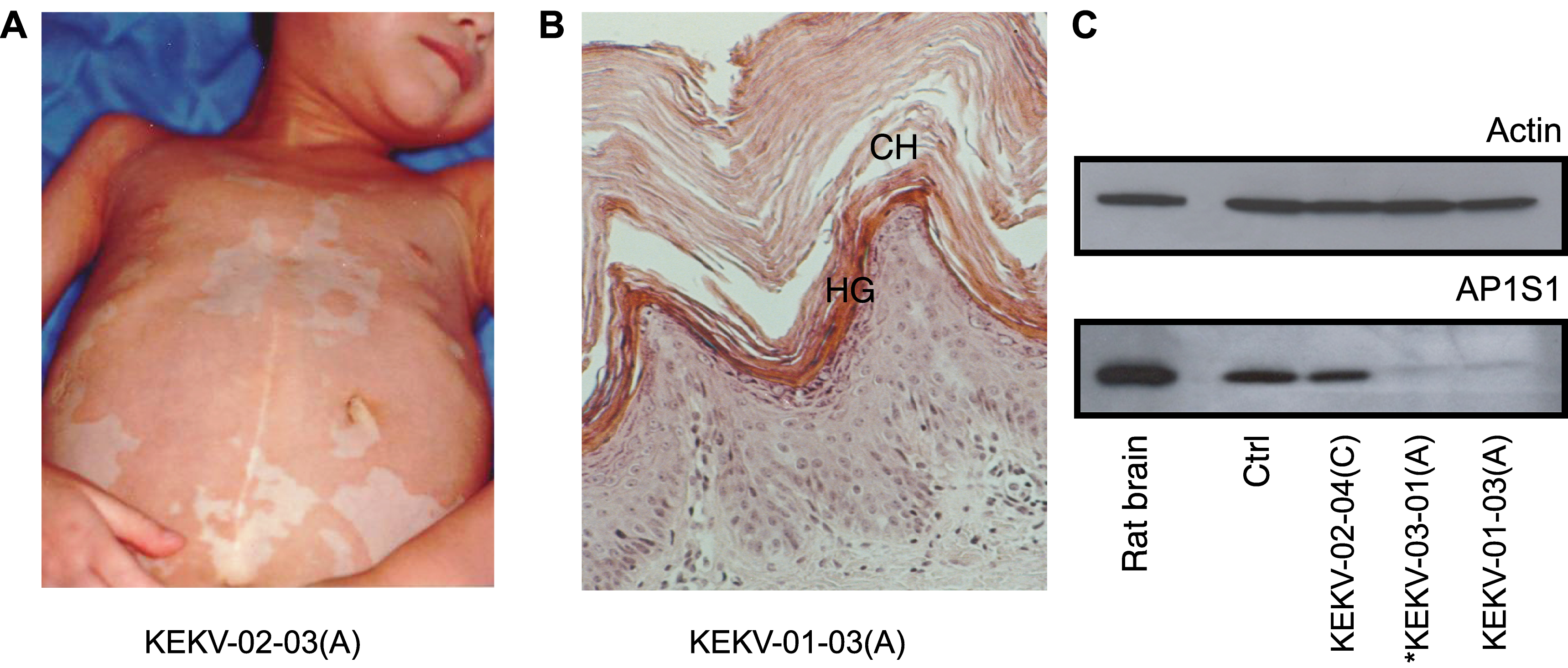

Supplement: Figure S1 — A) Typical erythrokeratodermia variabilis skin lesion. B) The histological analysis revealed an epidermal hyperplasia accompanied by compact hyperkeratosis (CH) and hypergranulosis (HG). C) Normalized western blot analysis of skin proteins indicates faint expression of Ap1s1 in affected individuals (A), probably because of partial expression of the isoform lacking three amino acids, compared to mutation carriers KEKV-02-04 (C) and control (Ctrl).Proteins were extracted from biopsies obtained from both lesional and non-lesional (*) skin in individual KEKV-01-03. (8.78 MB TIF) [file pgen.1000296.s001.tif]

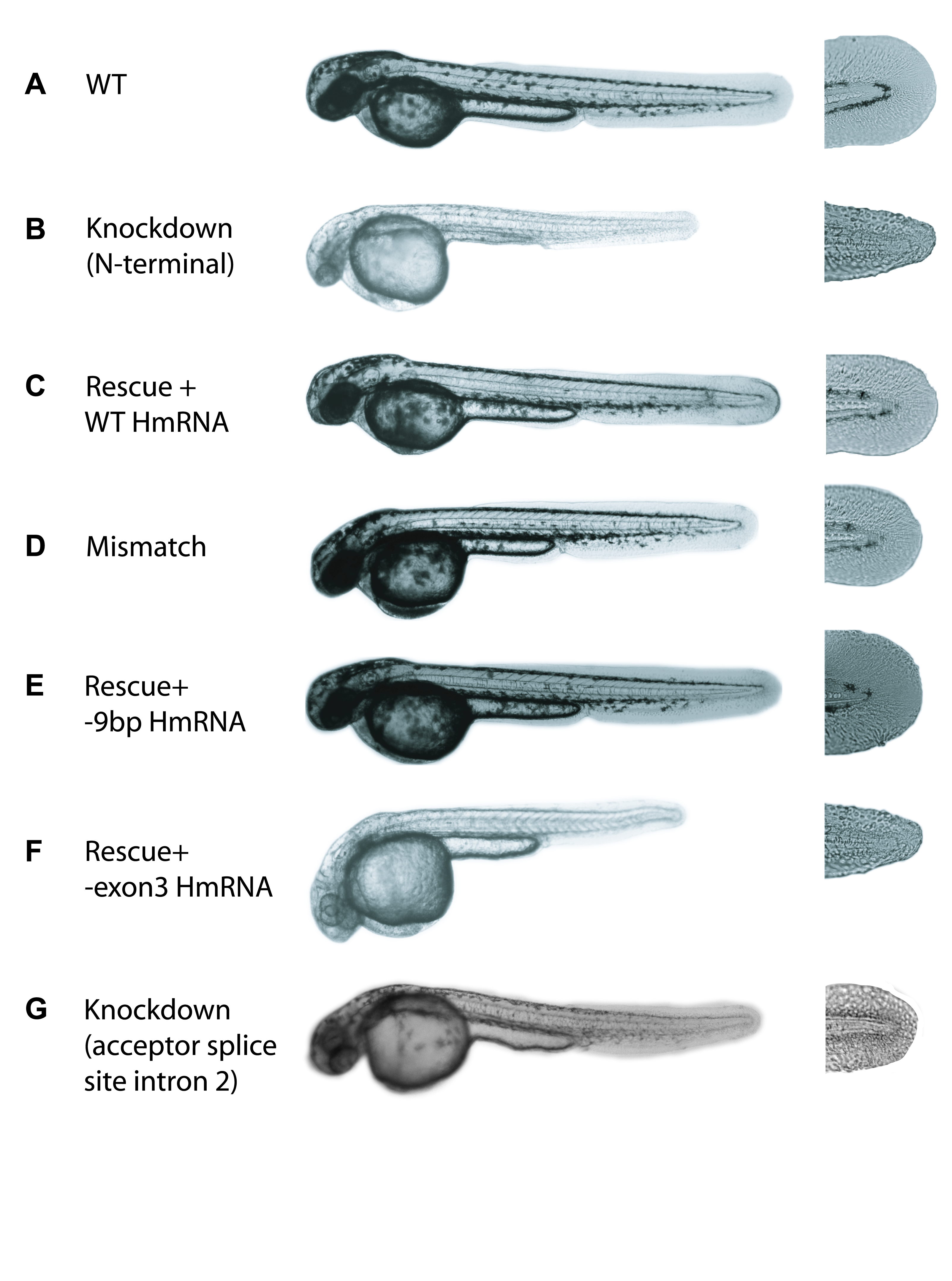

Supplement: Figure S2 — Representative transmitted light images of 48 hours-old zebrafish larvae illustrating the phenotype under different experimental conditions. (A) Wild type (WT), non injected larvae. (B) Knockdown larva, injected with AMO targeting the translation start site of Ap1s1 (N-terminal). (C) Larva rescued by co-injection of AMO and human WT mRNA (HmRNA). (D) Larva injected with a mismatch AMO. (E) Larva rescued by co-injection of AMO and -9bp Human mRNA. (F) larva co-injected with AMO and -exon3 HmRNA. (G) Knockdown larva, injected with AMO targeting the acceptor splice site (intron 2) of Ap1s1. (7.41 MB TIF) [file pgen.1000296.s002.tif]
