# Supplementary material for: Disruption of AP1S1, Causing a Novel Neurocutaneous Syndrome, Perturbs Development of the Skin and Spinal Cord
Source: PLoS Genet. 2008 Dec 5;4(12):e1000296. doi: 10.1371/journal.pgen.1000296 (PMC2585812; doi:10.1371/journal.pgen.1000296)
Supplement: Table S1 — Clinical features of affected individuals with MEDNIK. EKV: erythokeratodermia variabilis; VLCFA: very long chain fatty acids; NA: not available; mo: month. (0.03 MB DOC) [file pgen.1000296.s003.doc]

**Supplementary table 1**

| **Individual** | **Facial appearance** | **Cutaneous manifestations** | **Neurological manifestations** | **Gastro- intestinal manifestations** | **VLCFA** | **Other** |
| --- | --- | --- | --- | --- | --- | --- |
| KEKV02-03 | triangular, high forehead | EKV  ichthyosiform erythrodermia | psychomotor retardation, hypotonia, peripheral neuropathy, sensorineural deafness | congenital diarrhea | ↑C24+  ↑C26++ | cataracts |
| KEKV02-05 | NA | NA | NA | congenital diarrhea,  hepatic fibrosis, cholestasis | NA | cataracts, death  (7 mo) |
| KEKV01-03 | mongoloid, high forehead | EKV  icthyosiform erythrodermia | psychomotor retardation, hypotonia, peripheral neuropathy, sensorineural deafness | congenital diarrhea | ↑C24+  ↑C26++ |  |
| KEKV01-04 | NA | NA | NA | congenital diarrhea  icterus | NA | death  (16 days) |
| KEKV03-03 | mongoloid, high forehead | EKV  ichthyosiform erythrodermia | psychomotor retardation, hypotonia, peripheral neuropathy,  sensorineural deafness | congenital diarrhea, intestinal volvulus | ↑C24+  ↑C26++ | nephro-calcinosis |
| KEKV04-03 | mongoloid, high forehead | ichthyosis | psychomotor retardation, sensorineural deafness | congenital diarrhea, cholestasis, cirrhosis, microcolon | NA | death  (16 mo) |
| KEKV05-08 | NA | ichthyosis | hypotonia,  sensorineural deafness | congenital diarrhea, cholestasis, cirrhosis, jejunal atresia | ↑C24+  ↑C26++ | death  (18 mo) |
| KEKV05-09 | NA | ichthyosis | NA | congenital diarrhea, cholestasis, icterus |  | death  (2 mo) |
